# Sex Differences in the White Matter and Myelinated Fibers of APP/PS1 Mice and the Effects of Running Exercise on the Sex Differences of AD Mice

**DOI:** 10.3389/fnagi.2018.00243

**Published:** 2018-08-17

**Authors:** Chun-ni Zhou, Feng-lei Chao, Yi Zhang, Lin Jiang, Lei Zhang, Yan-min Luo, Qian Xiao, Lin-mu Chen, Yong Tang

**Affiliations:** ^1^Department of Histology and Embryology, Chongqing Medical University, Chongqing, China; ^2^Laboratory of Stem Cells and Tissue Engineering, Chongqing Medical University, Chongqing, China

**Keywords:** white matter, myelinated fibers, transgenic mouse model, sex differences, Alzheimer's disease, running exercise, stereology

## Abstract

Previous studies have suggested that changes in the white matter might play an important role in the pathogenic processes of Alzheimer's disease (AD). However, no study has investigated sex differences in these changes. Previous studies found that running exercise could delay both the decline in spatial learning and memory abilities as well as the changes in the white matter during early AD in male mice. However, whether exercise also has an effect on the changes in the white matter in female AD mice remains unknown. To address these questions, 6- and 10-month-old male and female APP/PS1 double transgenic AD mice were used. The 6-month-old male and female APP/PS1 double transgenic AD mice underwent a 4-month running exercise regime. The white matter volume and parameters of the myelinated fibers in the white matter of the 10-month-old exercised and non-exercised male and female AD mice were investigated using electron microscopy and stereological methods. There were no significant differences in the mean escape latencies between the male and female AD mice in the non-exercised groups, but after 4 months of treadmill exercise, the mean escape latencies of the female exercised AD mice had significantly shortened compared with those of the male exercised AD mice. The total white matter volume and most of the parameters of the myelinated fibers of the white matter in the female AD mice were significantly lower than those of the male AD mice. The total length of the myelinated fibers with diameters ranging from 0.6 to 0.7 μm, the axonal diameter of the myelinated fibers and the g-ratio of the myelinated fibers in the white matter of the exercised female AD mice were significantly increased compared with those of the non-exercised female AD mice. There were sex-specific differences in the white matter and myelinated fibers of white matter in the AD mice. Running exercise more effectively delayed the decline in spatial learning and memory abilities and delayed the changes in the myelinated fibers of the white matter in female transgenic mice with early AD than in male transgenic mice.

## Introduction

Alzheimer's disease (AD) is a progressive neurodegenerative disorder associated with cognitive decline. It has been reported that approximately 13% of individuals over the age of 65 years and 45% of individuals aged move than 85 years are diagnosed with AD (Liu et al., 2013; Alzheimer's Association, 2016). The total number of AD patients worldwide is estimated to equal 36 million, with the number of patients tripling by the year 2050 (Wimo and Prince, 2010). It is worth noting that both the prevalence and incidence of AD are greater among women than among men, and the discrepancy increases with increasing age (Andersen et al., 1999; Lobo et al., 2000). Taking Americans as an example, research has reported that two-thirds of the more than 5 million Americans living with dementia due to AD are women, and women account for approximately 65% of the 15 million unpaid caregivers of individuals with AD (Bouldin and Andresen, 2010). Barnes et al used postmortem samples from 141 older people to analyze the association between AD pathology and the clinical symptoms of AD and found that the link was significantly stronger in women than in men (Barnes et al., 2005). Men and women exhibit differences in the development and progression of AD. The prevalence and incidence of AD varied by sex and gender. There were also clear sex and gender-specific risk factors for AD. Ignoring these differences will impede research and treatment (Mielke et al., 2014). The area of gender differences in AD, although still largely unexplored, appeared to offer great promise for the future development of better strategies of intervention for patients (Musicco, 2009).

What might be the neural structural bases underlying the sex-specific differences in AD? The multimodality brain imaging results from Mosconi et al. indicated sex differences in the development of the AD endophenotype, suggesting that the preclinical AD phase was early in the female aging process and coincided with the endocrine transition of perimonopause (Mosconi et al., 2017). Women diagnosed with AD experiences a faster progression of hippocampal atrophy than did men, whereas men might be more likely to progress to AD in the presence of severe periventricular white matter hyperintensities (WMH) and reduced global cognitive performance (Mazure and Swendsen, 2016). Burke et al reported that the changes in hippocampal volumes affected the progression to or odds of probable AD (and MCI) more so among women than men, while changes in WMH affected the progression to MCI only among men, and the changes in WMH did not affected progression to probable AD among men or women (Burke et al., 2018).

Although many previous studies on AD have focused on gray matter changes in AD, many studies have also reported white matter changes in AD patients (Brun and Englund, 1986; Scheltens et al., 1995; Smith et al., 2000; Bronge et al., 2002). A previous imaging study revealed that the white matter volume of AD patients was decreased compared with that of healthy subjects (Bozzali et al., 2002). Previous studies have reported that the integrity of white matter is destroyed in AD patients (Bozzali et al., 2002; Huang et al., 2012). Kavcic et al. even found that white matter integrity is linked to cognitive function in AD patients (Kavcic et al., 2008). The pathological changes in the white matter of AD include axon damage, decreased myelin density, decreased myelin basic protein, the loss and breakdown of oligodendrocytes and microglia activation (Benes et al., 1991; Roher et al., 2002; Bartzokis, 2004; Wang et al., 2004; Sjöbeck et al., 2005, 2006). Bartzokis et al. showed that changes in the myelinated fibers are the most significant pathological change observed in the white matter in AD patients (Bartzokis et al., 2003, 2007; Bartzokis, 2004). Kruggel et al. (2017) analyzed longitudinal diffusion-weighted images in healthy and pathological aging and reported that white matter areas could reliably discriminate between patients with Althzimer's disease and healthy controls (Kruggel et al., 2017). Thus, a change in the myelinated fibers in white matter might play an important role in the pathogenic processes of AD. Gallart-Palau et al. reported that women with Alzheimer's disease and cerebrovascular disease exhibited greater severity alterations in the white matter than men (Gallart-Palau et al., 2016). However, Burke et al. reported that changes in WMH did not affect progression to probable AD among either men or women (Burke et al., 2018). Thus, it can be seen that studies on sex differences in the white matter of AD are ambiguous, and it is not clear whether sex differences exist in the myelinated fibers within the white matter in AD. The striking quantity and diversity of sex-related influences on brain function indicate that the still widespread assumption that sex influences are negligible cannot be justified and likely retards progress in the neuroscience field (Cahill, 2006). Therefore, it is important to investigate whether there are sex differences in the changes in white matter and the myelinated fibers within the white matter during early AD.

Many studies have suggested that physical exercise is a simple and economic life-style factor that might delay the onset and slow the progression of AD (Laurin et al., 2001; Hirsch, 2013) and physical exercise improves memory and cognitive function in transgenic AD mice (Nichol et al., 2009; Yuede et al., 2009). Our team previously found that 4 months of running exercise could delay the decline in spatial learning and memory ability, delay the progress of the changes in the white matter and the myelinated fibers in the white matter of early male APP/PS1 AD mice (Zhang et al., 2016). In female Tg CRND8 AD mice, Adlard et al. reported that 5 months of voluntary exercise enhanced the rate of learning (Adlard et al., 2005). Whether exercise also has an effect on changes in the white matter and the myelinated fibers within white matter in female AD mice remains unknown. If exercise has positive effects on the white matter and the myelinated fibers in white matter of early female AD mice, as it does for the early male AD, the more beneficial effect is unclear. Moreover, it is not clear whether exercise could alleviate the sex differences in the white matter and myelinated fibers in the white matter during early AD. To investigate all of these issues, in the present study, 10-month-old male and female APP/PS1 double transgenic AD mice were evaluated, and the sex differences in the white matter and myelinated fibers in the white matter of AD mice were investigated using electron microscopy and stereological methods. Then, 6-month-old male and female APP/PS1 double transgenic AD mice were subjected to a 4-month running exercise intervention, and the effects of running exercise on the sex differences in behaviors, white matter and myelinated fibers in the white matter of AD mice were investigated through behavioral tests, electron microscopy, and stereological methods.

## Materials and methods

### Animals

All APP/PS1 double transgenic mice *[heterozygous species B6C3-Tg (APPswe, PSEN1de9)85Dbo/J]* were provided by the Animal Model Institute of Nanjing University and reproduced in the Experimental Animal Center at Chongqing Medical University, P. R. China. At the end of the study, 2 male mice in male exercised group died. Therefore, two more male mice were provided in order to have seven mice per group at the end of the study. Seven male and seven female 10-month-old APP/PS1 transgenic mice were randomly selected and were regarded as the non-exercised groups. Fourteen male and 14 female 6-month-old APP/PS1 transgenic mice were also used. Seven male and seven female 6-month-old APP/PS1 transgenic mice were randomly selected and used as the exercised groups. The other seven male and seven female 6-month-old APP/PS1 transgenic mice were used as the non-exercised groups. The mice in the non-exercised groups were housed in controlled condition for 4 months without running. The mice in the exercised groups underwent a running exercise regime for 4 months. The mice were housed in controlled conditions (temperature 22 ± 2°C, lights on from 7:00 to 19:00, humidity 55%). Mouse chow and water were available *ad libitum*. All animal care and experimental protocols were approved by the Animal Care and Research Committee of Chongqing Medical University andwere in accordance with the guidelines of the National Institutes of Health Guide for the Care and Use of Laboratory Animals (NIH PublicationNo. 85-23).

### Running exercise

Male and female 6-month-old APP/PS1 transgenic mice(the exercised groups) were subjected to 4 months of running exercise training on a six-lane leveled motorized treadmill (SLY-TML, Beijing Sunny Instruments Co. Ltd., Beijing, P. R. China) at a speed of 10 m/min for 20 min/day and 5 days/week (Yuede et al., 2009). The first week corresponded to an adaptation period, during which the mice in the running group ran at a speed of 5 m/min for 10 min/day. After the first week, the mice in the running group continued to run at a speed of 10 m/min for 20 min/day.

### Morris water maze test

The Morris water maze (SLY-WMS, Beijing Sunny Instruments Co. Ltd., Beijing, P. R. China) was used to test spatial learning and memory abilities (Morris, 1984). The equipment included a stainless-steel circular pool with a diameter of 90 cm. The platform was made of transparent plastic with a diameter of 10 cm. The water of the pool was dyed white with milk. The temperature of the water was controlled at 22–25°C throughout the experiment. The pool was divided into four quadrants, and the mice were placed into the water at the points where the dividing lines of the quadrants intersected the pool wall. The first 6 days corresponded to the hidden platform test. The platform was hidden 1.0 cm below the water surface. The mice were first placed on the platform for 15 s. The mice were then placed into the water with their faces toward the wall and were allowed to search for the platform. If the mouse did not find the platform after 60 s, the mice were placed on the platform for 15 s and were then returned to their cage. On the seventh day, the mice performed the space exploration task. The hidden platform was removed, and the mice were placed into the water at the two intersections that were farthest away from the location where the platform had been. The mice were allowed to search for the platform in the pool for 60 s. The number of times the mouse crossed the position where the platform had been located was used as an indicator of the spatial cognition and memory abilities of the mouse. During the experiment, the time and trails were recorded by a camera system positioned above the pool (Chen et al., 2000).

### Tissue processing

After the Morris water maze, each mouse was anesthetized via intraperitoneal injection of 1% pentobarbital sodium (0.4 ml/100 g). The mouse's heart was exposed, a perfusion needle was inserted into the left ventricle, and the right atrial appendage was cut. The mouse was first perfused with saline solution and then perfusion-fixed with 2% paraformaldehyde (GuangFu Chemical Engineering Institute, TianJin, P. R. China) and 2.5% glutaraldehyde (Sinopharm Chemical Reagent Co., Ltd., Shanghai, P. R. China) in 0.1 M phosphate-buffered saline (PBS, pH 7.4). After perfusion, the brain was removed, and the olfactory bulb and cerebellum were dissected. The remainder of the brain was divided into two hemispheres. One of the two hemispheres from each mouse was randomly selected for the following experiment. Each hemisphere was cut into 1-mm-thick slices along the coronal direction. An average of 8–10 slices was obtained from each hemisphere. A point grid was randomly placed over the brain slices. From each hemisphere, three to four white matter blocks with a volume of 1 mm^3^ were sampled at the locations where the points of the grid corresponded to white matter. The sampled tissues were fixed in 4% glutaraldehyde solution for 2 h at 4°C andrinsed three times with 0.1 M PBS (pH 7.2). The tissue samples were then osmicated in 1% 0.1 M phosphate-buffered osmium tetroxide (Sinopharm Chemical Reagent Co., Ltd., Shanghai, P. R. China) at 4°C for 2 h. The blocks were gradually dehydrated though a 50, 70, and 90% ethanol (Chuandong Chemical Reagent Co. Ltd., ChongQing, P. R. China) series, a mixture of 90% ethanol and 90% acetone (Shanghai Chemical Reagent Main plant, Shanghai, P. R. China) and then 100% acetone. The tissue blocks, which were infiltrated with epoxy resin 618 (ChenGuang Chemical Industry, Sichuan, China), were first embedded in 5-mm spheres with epon. The spheres were subsequently rotated randomly on the table before being re-embedded. This method is known as isector (Nyengaard and Gundersen, 1992), which ensures that the distributions of nerve fibers in all directions have the same probability of being selected. One section with a thickness of 60 nm was cut from each epon block using an ultramicrotome (EM UC6, Leica, Germany) and was viewed under a transmission electron microscope (Hitachi-7500, Hitachi, Ltd., Japan). For each section, 10 fields of vision were randomly selected and photographed at a magnification of 8,000x. Twelve fields of vision were randomly chosen and photographed at a magnification of 20,000x.

### Estimation of white matter volume

**Figure 1:**
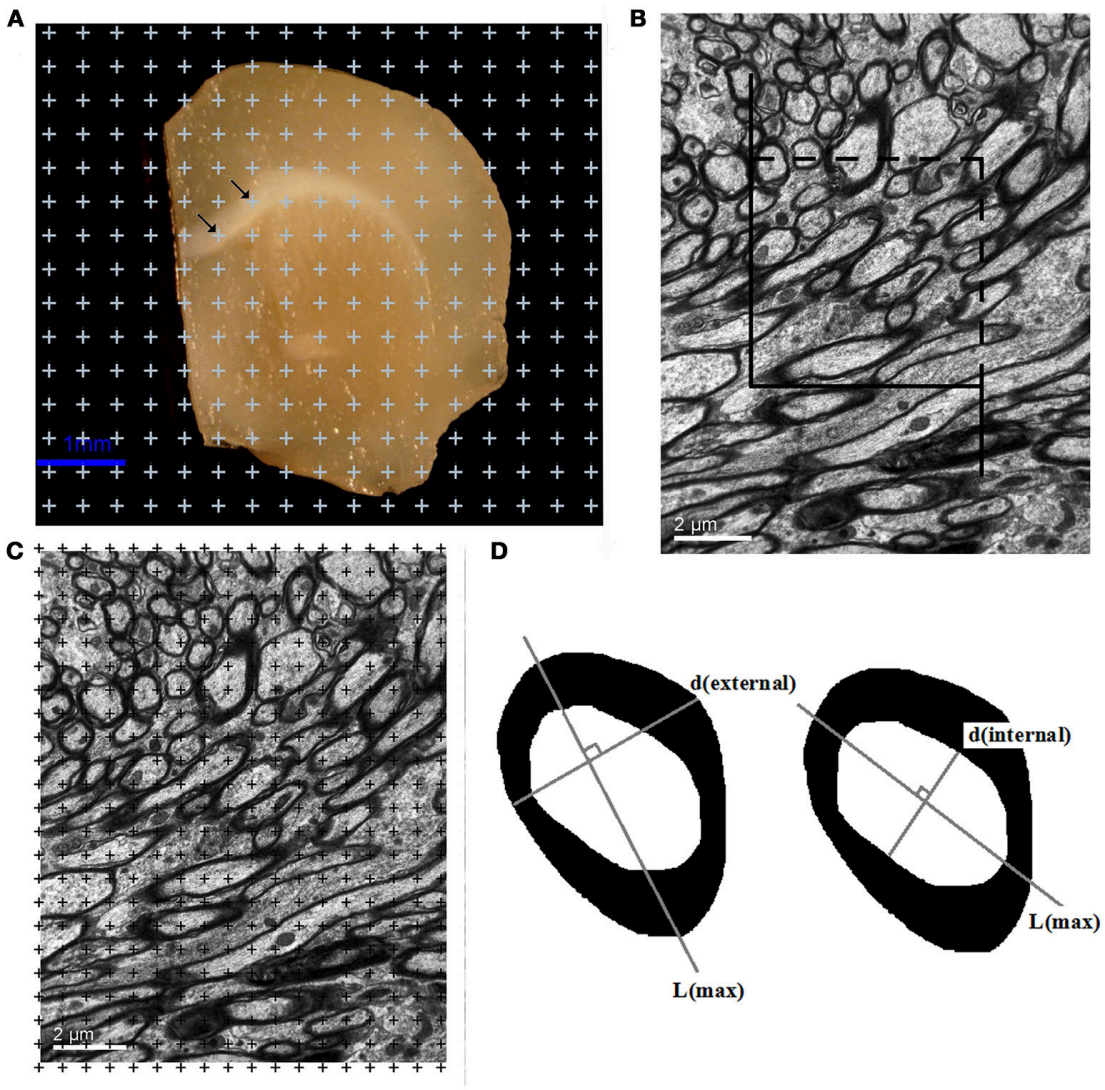
**(A)** A sample brain slice traced under a microscope at a magnification of 35x. All points that overlaid the white matter were counted, as indicated by the arrows. Bar = 1 μm. **(B)** A transparent counting grid was randomly overlaid on the photographs (8,000x magnification). The number of points that touched the white matter, the myelinated fibers, the myelin sheaths, and the axons in the white matter were counted. Bar = 2 μm. **(C)** An unbiased counting frame was randomly superimposed on the captured photographs at a magnification of 8,000x. The myelinated nerve fiber profiles inside the counting frame or touching the top and right borders were counted. The myelinated nerve fiber profiles touching the left line, the bottom line and the extensions of the right and left lines were not counted. Bar = 2 μm. **(D)** Left: The outer diameter [d(external)] of the myelinated fibers sampled with the unbiased counting frame was estimated by measuring the myelinatedfiber profile diameter perpendicular to the longest axis of the myelinated fiber (L). Right: The inner diameter [d(internal)] of the myelinated fibers sampled with the unbiased counting frame was estimated by measuring the axonal profile diameter perpendicular to the longest axis of the axon (L).

Each brain slice was photographed, and the point grid with equidistant points was randomly superimposed on each photograph of the brain slice. The total number of points that overlaid white matter was counted. The total volume of the white matter, V(w) (Figure 1A), was calculated using Cavalieri's principle (Tang and Nyengaard, 1997; Tang et al., 1997):

(1) $$\begin{array}{l} \text{V}\left(\text{w}\right)=\text{t}\times \text{a}\left(\text{p}\right)\times \sum \text{P}\left(\text{w}\right) \end{array}$$

where V(w) is the total volume of the white matter, t is the slice thickness (1 mm) and ∑P(w) is the total number of points that hit white matter for each mouse.

### Estimation of the total length of the myelinated fibers in white matter

An unbiased counting frame (Gundersen, 1977) was randomly superimposed on the captured photographs at a magnification of 8,000x. The myelinated nerve fiber profiles inside the counting frame and touching the top and right borders were counted. The myelinated nerve fiber profiles touching the left line, the bottom line and the extensions of the right and left lines were not counted (Figure 1B). The total length of the myelinated fibers, L(mf, w), was calculated as follows (Gundersen et al., 1988; Tang et al., 1997, 2003):

(2) $$\begin{array}{l} L\left(mf,w\right)=2\times \frac{\sum Q\left(mf/w\right)}{\sum A} \end{array}$$

where L(mf/w) is the total length of the myelinated fibers in the white matter, ∑Q(mf) is the total number of myelinated fiber profiles counted in the white matter per mouse, ∑A is the total area of the unbiased counting frames used per mouse, and V(w) is the total volume of the white matterper mouse.

### Estimation of the total volumes of the myelinated fibers, the myelin sheaths, and the axons in the white matter

A transparent counting grid was randomly placed over the photographs. The number of points that hit the white matter, ∑P(w), the myelinated fibers, ∑P(mf), the myelin sheaths, ∑P(ms), and the axons, ∑P(axon), in the white matter was counted (Figure 1C). The volume density of myelinated fibers, Vv(mf/w), the volume density of the myelin sheath, Vv(ms/w), and the volume density of axons, Vv(axon/w), in white matter were then calculated with the following formulas (Gundersen et al., 1988; Tang et al., 1997, 2003):

(3) $$\begin{array}{l} \text{Vv}\left(\text{mf}/\text{w}\right)=\frac{\sum P\left(mf\right)}{\sum P\left(w\right)} \end{array}$$

(4) $$\begin{array}{l} \text{Vv}\left(\text{ms}/\text{w}\right)=\frac{\sum P\left(ms\right)}{\sum P\left(w\right)} \end{array}$$

(5) $$\begin{array}{l} \text{Vv}\left(\text{axon}/\text{w}\right)=\frac{\sum P\left(axon\right)}{\sum P\left(w\right)} \end{array}$$

The total volumes of the myelinated fibers, V(mf, w), the myelin sheaths, V(ms, w), and the axons, V(axon, w), in the white matter were subsequently estimated as follows:

(6) $$\begin{array}{l} \text{V}\left(\text{mf},\text{w}\right)=Vv\left(mf/w\right)\times V\left(w\right) \end{array}$$

(7) $$\begin{array}{l} \text{V}\left(\text{ms},\text{w}\right)=Vv\left(ms/w\right)\times V\left(w\right) \end{array}$$

(8) $$\begin{array}{l} \text{V}\left(\text{axon},\text{w}\right)=Vv\left(axon/w\right)\times V\left(w\right) \end{array}$$

### Estimation of the inner and outer diameters of the myelinated fibers in the white matter

Transmission electron microscopy images were randomly captured with a magnification of 20,000x. The unbiased counting frame (Tang et al., 1997) was randomly superimposed on the randomly captured images. All of the myelinated fibers sampled with the unbiased counting frame were used for the diameter measurements. First, the longest axis of the axons of the myelinated fibers and the longest axis of the myelinated fibers were drawn. The inner and outer diameters of the myelinated fibers were then obtained from the longest distance perpendicular to the longest axis of the axons and the longest distance perpendicular to the longest axis of the myelinated fibers (Gundersen, 1977) (Figure 1D).

### Statistical analyses

All statistical analyses were performed with SPSS19.0 (ver. 19.0, SPSS Inc., Chicago, IL, USA). The Morris water maze data between the male and female non-exercised groups and between the male and female exercised groups from day 1 to day 6 were analyzed by repeated-measures analysis of variance (ANOVA). The Morris water maze data between the male and female no-exercised groups and between the male and female exercised groups on day 7 were analyzed using one-way ANOVA. The Shapiro-Wilk test was used to evaluate whether the group means of the stereological data were normally distributed. When normality assumptions were satisfied, the stereological data between the male and female no-exercised groups, the stereological data between the male and female exercised groups, the stereological data between the male no-exercised group and exercised group, and the stereological data between the female no-exercised group and exercised group were analyzed using an unpaired, two-tailed Student's *t*-test. When normality assumptions were not satisfied, the stereological data between the male and female no-exercised groups, the stereological data between the male and female exercised groups, the stereological data between the male no-exercised group and exercised group, and the stereological data between the female no-exercised group and exercised group were analyzed using non-parametric tests. The mean difference was considered significant if *p* < 0.05. The data between the male non-exercised group and the exercised group were published to show the effects of running exercise on the white matter and the myelinated fibers in the white matter of early AD mice (Zhang et al., 2016).

## Results

### Morris water maze test

**Figure 2:**
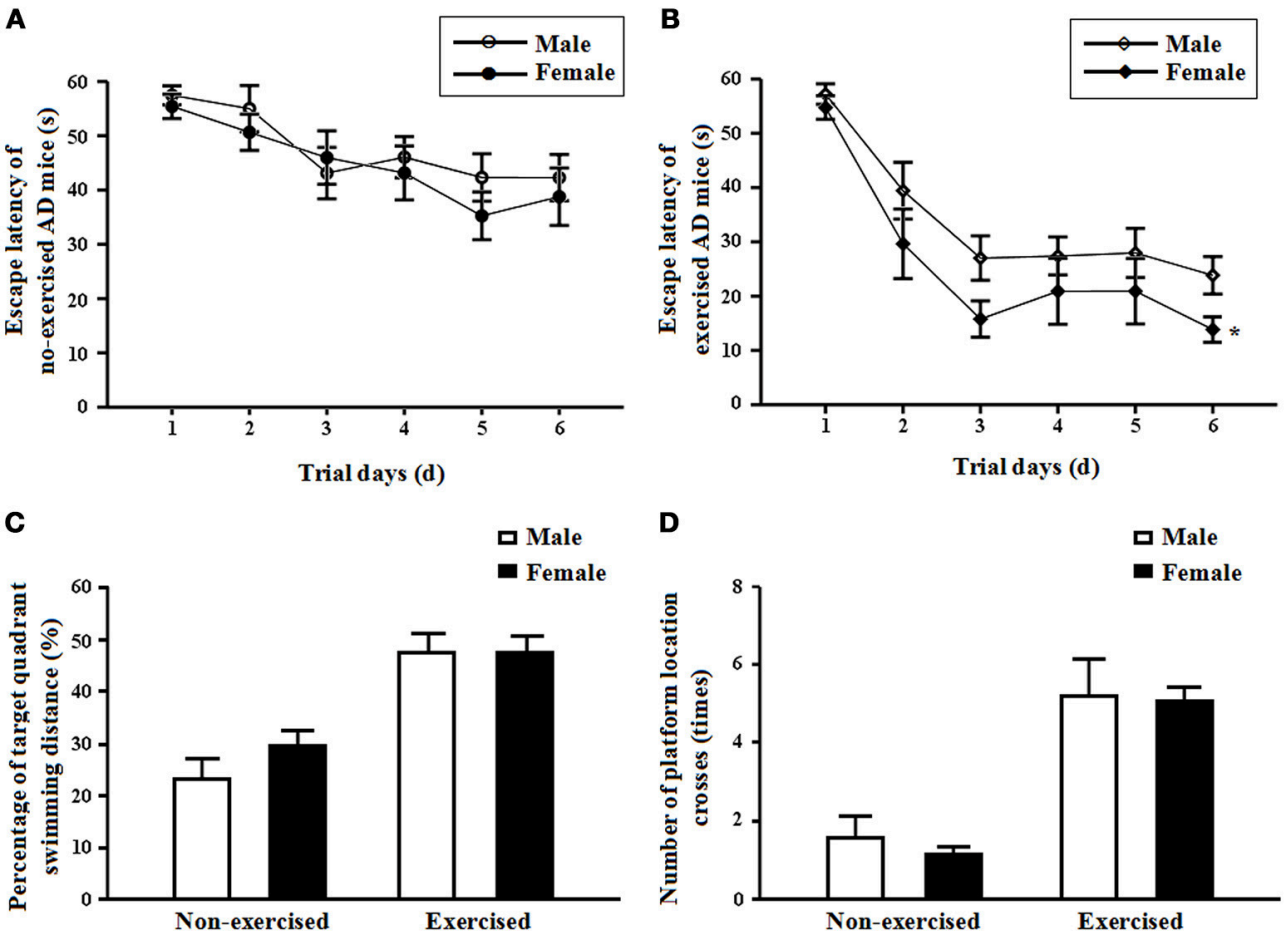
**(A)** Mean escape latencies of the male and femalenon-exercisedAPP/PS1 transgenic mice from days 1 to 6. Each point represents the mean ± standard error of the mean (SEM) of each group for trials 1-4 conducted on the corresponding day. **(B)** Mean escape latencies of the male and female exercised APP/PS1 transgenic mice from days 1 to 6. Each point represents the mean ± standard error of the mean (SEM) of each group for trials 1–4 conducted on the corresponding day. ^*^p < 0.05. (**C)** Percentages of target quadrant swimming distances on day 7 obtained for the male and female non-exercised or exercised APP/PS1 transgenic mice (mean ± SD). **(D)** Numbers of platform location crosses on day 7 obtained for the male and female non-exercised or exercised APP/PS1 transgenic mice (mean ± SD).

In the non-exercised groups, there were no significant differences in the mean escape latencies between the male and female AD mice (*p* = 0.49, Figure 2A). After 4 months of treadmill exercise, the mean escape latencies of the female exercised AD mice were significantly shortened compared with those of the male exercised AD mice (*p* = 0.02, Figure 2B).

In the non-exercised groups, there were no significant differences in the percentage of target quadrant swimming distance and in the number of platform location crosses between the male and female AD mice (*p* = 0.2 and *p* = 0.47, Figures 2C,D). After 4 months of treadmill exercise, there were no significant differences in the percentage of target quadrant swimming distance and in the number of platform location crosses between the male and female exercised AD mice (*p* = 0.97 and *p* = 0.89, Figures 2C,D).

### Stereological results

#### White matter volume

**Figure 3:**
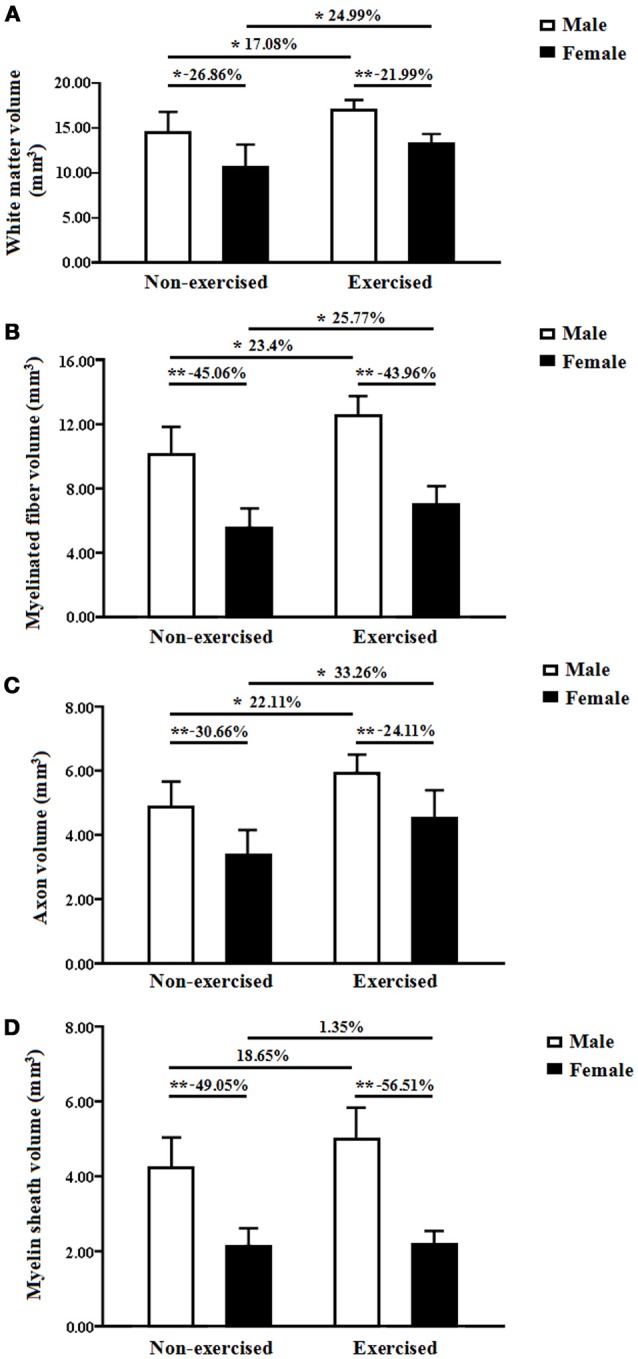
**(A)** Total white matter volumes of the male and female non-exercised or exercised APP/PS1 transgenic mice. ^*^p < 0.05, ^**^p < 0.01. **(B)** Total volumes of myelinated fibers of the male and female non-exercised or exercisedAPP/PS1 transgenic mice. ^*^p < 0.05, ^**^p < 0.01. **(C)** Axonal volumes of the myelinated fibers of the male and female non-exercised or exercised APP/PS1 transgenic mice. ^*^p < 0.05, ^**^p < 0.01. **(D)** Total volumes of the myelin sheaths of the male and female non-exercised or exercised APP/PS1 transgenic mice. ^**^p < 0.01.

In the non-exercised groups, the total volume of white matter in the female AD mice was significantly smaller by 26.86%than that in the male AD mice (*p* = 0.01, Figure 3A). After 4 months of treadmill exercise, the total volume of white matter in the exercised AD mice was significantly increased compared with that in the non-exercised AD mice, and this finding was found for both the male (increased by 17.08%) and female groups (increased by 24.99%) (*p* = 0.01 and *p* = 0.04, Figure 3A). After 4 months of treadmill exercise, the total volume of white matter in the female exercised AD mice was significantly smaller (21.99% smaller)than that in the male exercised AD mice (*p* = 0.00, Figure 3A).

#### Total volume of myelinated fibers

In the non-exercised groups, the total volume of the myelinated fibers within the white matter in the female AD mice was significantly smaller (45.06% smaller) than that in the male AD mice (*p* < 0.001, Figure 3B). After 4 months of treadmill exercise, the total volume of the myelinated fibers within the white matter in the exercised AD mice was significantly increased compared with that in the non-exercised AD mice, and this finding was obtained for both the male groups (increased by 23.4%) and the female groups (increased by 25.77%) (*p* = 0.01 and *p* = 0.04, Figure 3B). After 4 months of treadmill exercise, the total volume of the myelinated fibers within the white matter in the female exercised AD mice was significantly smaller (43.96%smaller) than that in the male exercised AD mice (*p* = 0.00, Figure 3B).

#### Axonal volume of the myelinated fibers

In the non-exercised groups, the axonal volume of the myelinated fibers within the white matter in the female AD mice was significantly smaller (30.66% smaller) than that in the male AD mice (*p* = 0.00, Figure 3C). After 4 months of treadmill exercise, the axonal volume of the myelinated fibers within the white matter in the exercised AD mice was significantly increased compared with that in the non-exercised AD mice, and this finding was obtained for both the male groups (increased by 22.11%) and the female groups (increased by 33.26%) (*p* = 0.02 and *p* = 0.03, Figure 3C). In addition, the axonal volume of the myelinated fibers within the white matter in the female exercised AD mice was significantly smaller (24.11% smaller) than that in the male exercised AD mice (*p* = 0.01, Figure 3C).

#### Total volume of the myelin sheaths

In the non-exercised groups, the total volume of the myelin sheaths of the white matter in the female AD mice was significantly smaller (49.05% smaller) than that in the male AD mice (*p* < 0.001, Figure 3D). After 4 months of treadmill exercise, there were no significant differences in the total volume of the myelin sheaths of the white matter between the non-exercised and exercised AD mice, and this finding was observed with both the male and the female groups(*p* = 0.11 and *p* = 0.90, Figure 3D). However, the total volume of the myelin sheaths of the white matter in the female exercised AD mice was significantly smaller (56.51% smaller) than that in the male exercised AD mice (*p* < 0.001, Figure 3D).

#### Total length of myelinated fibers

**Figure 4:**
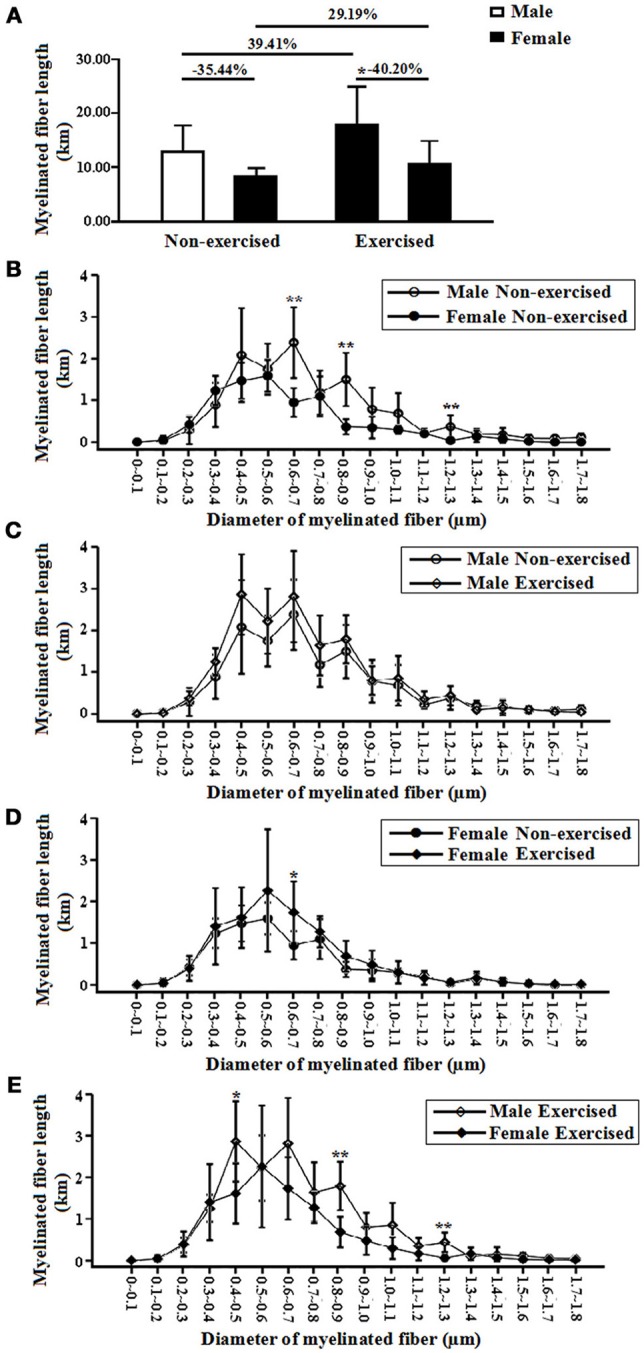
**(A)** Total lengths of myelinated fibers of the male and female non-exercised or exercised APP/PS1 transgenic AD mice. ^*^p < 0.05. **(B)** Log-scale absolute size distributions of the myelinated fiber diameters in the white matter of the male non-exercised AD mice (○) and female non-exercised mice (•). ^**^p < 0.01. The class width is 0.1 μm. The error bars indicate the SD. **(C)** Log-scale absolute size distributions of the myelinated fiber diameters in the white matter of the male non-exercised AD mice (○) and male exercised mice (◇). The class width is 0.1 μm. The error bars indicate the SD. **(D)** Log-scale absolute size distributions of the myelinated fiber diameters in the white matter of the female non-exercised AD mice (•) and female exercised mice (♦). ^*^p < 0.05. The class width is 0.1 μm. The error bars indicate the SD. **(E)** Log-scale absolute size distributions of the myelinated fiber diameters in the white matter of the male exercised AD mice (◇) and female exercised mice (♦). ^**^p < 0.01. The class width is 0.1 μm. The error bars indicate the SD.

In the non-exercised groups, the total length of the myelinated fibers within the white matter in the female AD mice was significantly shortened (35.44% shorter) compared with that in the male AD mice (*p* = 0.05, Figure 4A).The total lengths of the myelinated fibers with diameters ranging from 0.6 to 0.7, 0.8 to 0.9, and 1.2 to 1.3 μm in the female AD mice were significantly shorter than those of the male AD mice (*p* = 0.00, *p* = 0.00, and *p* = 0.00, Figure 4B).

After 4 months of treadmill exercise, there were no significant differences in the total lengths of the myelinated fibers within the white matter between the non-exercised and exercised AD mice in either the male or the female groups (*p* = 0.15 and *p* = 0.19, Figure 4A).In the male groups, there were no differences in the absolute size distributions of the myelinated fiber diameters in the white matter between the non-exercised and exercised AD mice (Figure 4C). However, in the female groups, the total length of the myelinated fibers with diameters ranging from 0.6 to 0.7 μm in the exercised AD mice was significantly longer than that of the non-exercised AD mice (*p* = 0.02, Figure 4D).

In addition, the total length of the myelinated fibers within the white matter in the female exercised AD mice was significantly smaller (40.20% smaller) than that in the male exercised AD mice (*p* = 0.04, Figure 4A). The total lengths of the myelinated fibers with diameters ranging from 0.4 to 0.5, 0.8 to 0.9, and 1.2 to 1.3 μm in the female AD mice were significantly shorter than those of the male AD mice (*p* = 0.02, *p* = 0.00, and *p* = 0.00, Figure 4E).

### Diameters of myelinated fibers

#### External diameter of myelinated fibers

**Figure 5:**
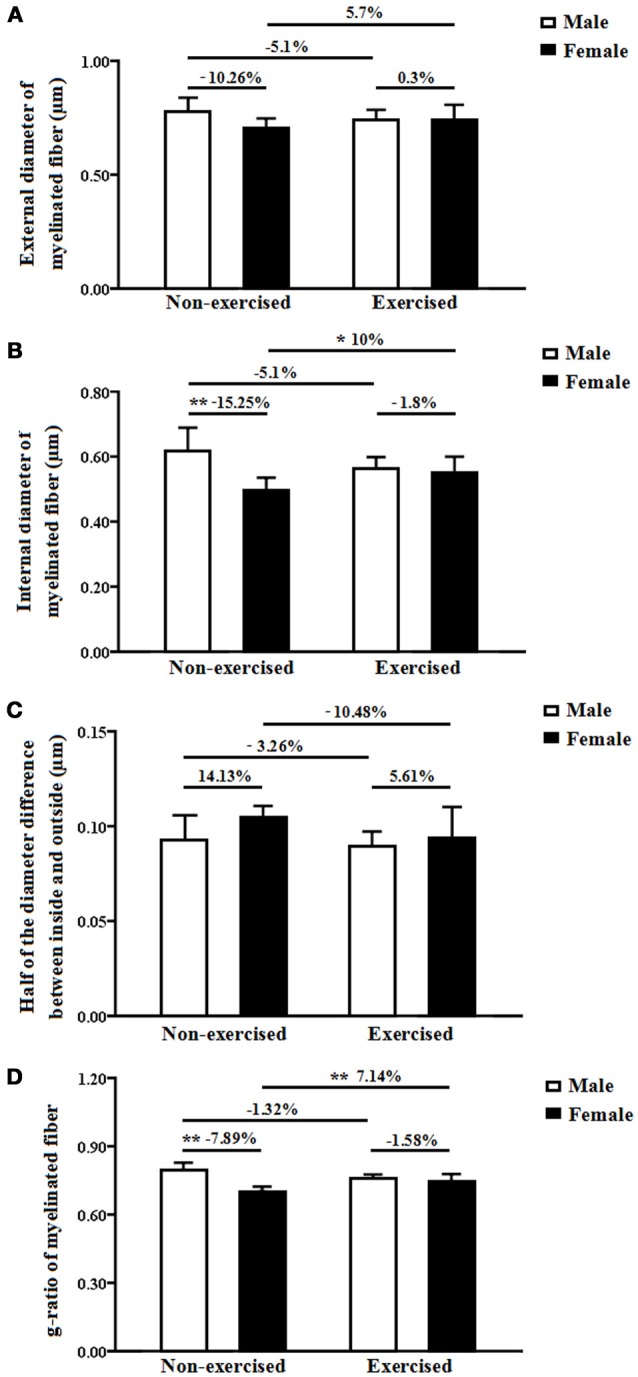
**(A)** External diameters of the myelinated fibers within the white matter in male and female non-exercised or exercised AD mice. **(B)** Internal diameters of myelinated fibers within the white matter in male and female non-exercised or exercised AD mice. ^*^p < 0.05, ^**^p < 0.01. **(C)** Half-differences between the inside and outside myelinated fiber diameters within the white matter in male and female non-exercised or exercised AD mice. **(D)** G-ratios of myelinated fibers in the white matter of male and female non-exercised or exercised AD mice. ^**^p < 0.01.

The mean external diameter of the myelinated fibers indicates the myelinated fiber diameter, including the diameters of the axon and the myelin sheath. In the non-exercised groups, there was no significant difference in the external diameter of the myelinated fibers within the white matter between the female and male AD mice (*p* = 0.06, Figure 5A). After 4 months of treadmill exercise, there were no significant differences in the external diameters of the myelinated fibers within the white matter between the non-exercised and exercised AD mice, and this finding was found for both the male and the female groups (*p* = 0.32 and *p* = 0.29, Figure 5A). In addition, there was no significant difference in the external diameter of the myelinated fibers within the white matter between the male exercised AD mice and the female exercised AD mice (*p* = 0.96, Figure 5A). The external diameter of the myelinated fibers might be, in part, affected by the regularity of the myelinated fibers. The current results showing that the axonal volume of the myelinated fibers and the total volume of the myelin sheaths within the white matter in the female AD mice were significantly smaller than those in the male AD mice, but the fact that there was no significant difference in the external diameters between the female and male AD mice suggests that total volume is a more precise parameter than diameter.

#### Internal diameter of myelinated fibers

The internal diameter of the myelinated fibers indicates the axonal diameter of the myelinated fibers. In the non-exercised groups, the internal diameter of the myelinated fibers within the white matter in the female AD mice was significantly smaller (15.25%smaller) than that in the male AD mice (*p* = 0.00, Figure 5B). After 4 months of treadmill exercise, there was no significant difference in the internal diameter of the myelinated fibers within the white matter between the non-exercised and exercised AD mice in the male groups (*p* = 0.32, Figure 5B); however, in the female groups, the internal diameter of the myelinated fibers within the white matter in the exercised AD mice was significantly increased (10% increased) compared with that in the non-exercised AD mice (*p* = 0.04, Figure 5B). In addition, there was no significant difference in the internal diameter of the myelinated fibers within the white matter between the male exercised AD mice and the female exercised AD mice (*p* = 0.66, Figure 5B).

#### Half-difference between the inside and outside myelinated fiber diameters

The half-difference between the inside and outside myelinated fiber diameters reflects the thickness of the myelin sheaths. In the non-exercised groups, there was no significant difference in the half-difference between the inside and outside myelinated fiber diameters between the male and female AD mice (*p* = 0.05, Figure 5C). After 4 months of treadmill exercise, there were no significant differences in the half-differences between the inside and outside myelinated fiber diameters between the non-exercised and exercised AD mice, and this finding was obtained for both the male and the female groups (*p* = 0.64 and *p* = 0.139, Figure 5C). In addition, there was no significant difference in the half-differences between the inside and outside myelinated fiber diameters between the male exercised AD mice and the female exercised AD mice (*p* = 0.53, Figure 5C).

#### G-ratio of myelinated fibers

The g-ratio of the myelinated fibers is equal to the ratio of the external to the internal diameters of the myelinated fibers. In the non-exercised groups, the g-ratio of the myelinated fibers within the white matter in the female AD mice was significantly smaller (7.89% smaller) than that in the male AD mice (*p* = 0.00, Figure 5D). After 4 months of treadmill exercise, there was no significant difference in the g-ratio of the myelinated fibers within the white matter between the non-exercised and exercised AD mice in the male groups(*p* = 0.82, Figure 5D); however, in the female groups, the g-ratio of the myelinated fibers within the white matter in the exercised AD mice was significantly increased (7.14% increased) compared with that in the non-exercised AD mice (*p* = 0.01, Figure 5D). In addition, there was no significant difference in the g-ratio of the myelinated fibers within the white matter between the male exercised AD mice and the female exercised AD mice (*p* = 0.4, Figure 5D).

## Discussion

Previous studies have shown that men and women exhibit differences in the development and progression of AD due to sex differences in the half-differences between the inside and outside myelinated fiber diameters (Andersen et al., 1999; Lobo et al., 2000; Barnes et al., 2005; Bouldin and Andresen, 2010). However, the mechanism underlying the sex-specific differences in AD is not well understood. A large number of previous studies have reported white matter changes in AD patients (Brun and Englund, 1986; Scheltens et al., 1995; Smith et al., 2000; Bronge et al., 2002), which might be closely related to the decline of cognitive function in AD patients (Bozzali et al., 2002; Kavcic et al., 2008; Huang et al., 2012). In our previous study, we found that the learning and memory abilities in 10-month-old APP/PS1 transgenic AD mice were significantly decreased compared with those of 10-month-old wild-type mice. In this study, we used male and female 10-month-old APP/PS1 transgenic AD mice to quantify the total volumes of white matter using stereological methods. Our results showed that the total volume of white matter in female AD mice was significantly smaller than that in the male AD mice, indicating the existence of a sex-specific difference in the white matter volume of AD mice. It is known that the main component of white matter is myelinated fibers. Previous studies have shown that changes in the myelinated fibers are the most significant pathological changes observed in the white matter of AD patients (Bartzokis et al., 2003; Bartzokis, 2004), but whether sex differences are present in the myelinated fibers in white matter during AD is unclear. In the current study, we investigated the relevant parameters of the myelinated fibers in the white matter of male and female AD mice and found that the values of most parameters in the female AD mice were significantly smaller than those in the male AD mice. First, although there was no significant difference in the mean diameter of the myelinated fibers (i.e., the external diameter of the myelinated fibers) of the white matter between male and female AD mice, the total volume of the myelinated fibers and the total length of the myelinated fibers within the white matter in the female AD mice were significantly lower than those obtained for the male AD mice. These results indicated that the existence of a sex-specific difference in the myelinated fibers in the white matter in AD mice. In addition, the substantial differences in the absolute distributions of the fiber diameters clearly illustrated sex-specific differences in the myelinated fibers with diameters ranging from 0.6 to 0.7, 0.8 to 0.9, and 1.2 to 1.3 μm in AD mice. Second, the axon volume of the myelinated fibers and the axonal diameter of the myelinated fibers (i.e., the internal diameter of the myelinated fibers) in the female AD mice were significantly lower than those in the male AD mice, indicating a sex-specific difference in the axons of the myelinated fibers in the white matter in AD mice. Third, the total volume of the myelin sheaths of the white matter in the female AD mice was significantly smaller than that in the male AD mice, but there was no significant difference in the half-difference between the inside and outside myelinated fiber diameters (reflecting the thickness of the myelin sheaths) of the white matter between the male and female AD mice. These results indicated the existence of a sex-specific difference in the total volume of the myelin sheaths but not in the thickness of the myelin sheaths within the white matter of AD mice. Finally, the g-ratio of the myelinated fibers within the white matter in the female AD mice (0.70) was significantly lower than that in male AD mice (0.76). Myelin provides insulation to axons and makes saltatory conduction possible; therefore, to a certain extent, the g-ratio reflects the rate of conduction along nerve fibers (Peters, 2009). Our results suggested a sex-specific difference in the rate of conduction along myelinated fibers in the white matter in AD mice. All of the above results suggested sex-specific differences in the white matter and myelinated fibers in the white matter in AD mice, which might provide an important scientific basis for understanding the gender differences in AD. The low values of the white matter volume and the relevant parameters of the myelinated fibers in the white matter in female AD mice might be an important structural basis for the high prevalence and incidence of AD among females.

Exercise has received widespread attention as a simple and affordable behavioral intervention. A large number of studies have shown that running exercise can improve cognitive ability and the primary functions of AD patients and AD mice (Cho et al., 2003; Adlard et al., 2005; Marx, 2005; Yu et al., 2006; Cotman and Berchtold, 2007; Pérez and Cancela Carral, 2008). Overwhelming evidence has revealed that running exercise can delay declines in spatial learning and memory ability in both female and male transgenic mouse models of AD (Adlard et al., 2005; Cotman and Berchtold, 2007). Our team previously found that a 4-month running exercise protocol could delay the cognitive decline of male APP/PS1 AD mice (Zhang et al., 2016). Our current study further investigated the effects of running exercise on female AD mice. In our current study, we found no significant differences in the mean escape latencies between male and female AD mice in the non-exercised groups, but after 4 months of treadmill exercise, the mean escape latencies of the female exercised AD mice were significantly shortened compared with those of the male exercised AD mice. Our current results suggested that running exercise delays the decline in spatial learning and memory abilities in early female transgenic AD mice more effectively than in early male transgenic AD mice.

As discussed above, sex-specific differences were identified in the white matter and myelinated fibers in AD mice. Therefore, we further investigated the effects of running exercise on the white matter and myelinated fibers within the white matter in male and female AD mice. We found that running exercise could prevent white matter atrophy and protect the myelinated fibers of white matter in both female and male transgenic mouse models of AD. Based on our results, running exercise had a greater beneficial effect on the white matter and the myelinated fibers of white matter in female AD mice than in male AD mice. Particularly in the female groups, the total length of the myelinated fibers with diameters ranging from 0.6 to 0.7 μm in the white matter of the exercised AD mice was significantly increased compared with the non-exercised AD mice, whereas there were no significant differences in the absolute size distributions of the myelinated fiber diameters in the white matter between the non-exercised and exercised AD mice in the male groups. These results indicated that running exercise could delay the loss of myelinated fibers with diameters ranging from 0.6 to 0.7 μm in the white matter only in female AD mice. Furthermore, in the female groups, the axonal diameter of the myelinated fibers and the g-ratio of the myelinated fibers in the white matter of the exercised AD mice were significantly increased compared with those of the non-exercised AD mice, whereas there were no differences in the axonal diameter of the myelinated fibers and the g-ratio of the myelinated fibers in the white matter between the non-exercised and exercised male AD mice. These results indicated that the running exercise had a greater beneficial effect on the axonal diameter of the myelinated fibers and the g-ratio of the myelinated fibers in the white matter of the female AD mice than in the male AD mice. After 4 months of treadmill exercise, the sex-specific differences still existed in the white matter and the myelinated fibers of AD mice. However, the sex differences in the total volume of white matter and most of the parameters of the myelinated fibers in the white matter (such as the total volume of the myelinated fibers, the axonal volume of the myelinated fibers, the mean diameter of the myelinated fibers, the axonal diameter of the myelinated fibers, the thickness of the myelin sheaths, and the g-ratio of the myelinated fibers) were decreased by running exercise in AD mice. In contrast, the sex differences in the total volume of myelin sheaths and the total length of the myelinated fibers in the white matter were increased by running exercise in AD mice. Although the sex difference in the total length of the myelinated fibers in the white matter was increased by running exercise in AD mice, the running exercise could delay the loss of myelinated fibers with diameters ranging from 0.6 to 0.7 μm in the white matter in female AD mice and could reduce the sex difference in the total length of the myelinated fibers with diameters ranging from 0.6 to 0.7 μm in the white matter of AD mice. Taken together, the results indicate that running exercise could more effectively protect the myelinated fibers of white matter in female transgenic mice with early AD than in male transgenic mice and could decrease the sex-specific differences in the white matter and the myelinated fibers of white matter in AD mice.

In the current study, we found that running exercise delayed the spatial learning and memory ability decline in the early transgenic mouse model of AD mice more effectively in females than in males. Moreover, we found that the effects of the running exercise on the white matter and the myelinated fiber in the white matter of female AD mice were more beneficial than those observed in male AD mice. What might be the reasons for the different effects of exercise on the female and male AD mice? We speculate that one possible explanation for these sex differences might be related to estrogen. The relationship between low estrogen and AD risk is also supported by the findings on the interactions between dementia risk and surgical menopause by oophorectomy and/or hysterectomy; specifically, dementia risk is significantly increased by surgically induced menopause (Rocca et al., 2007; Phung et al., 2010; Bove et al., 2014). Women with AD exhibited significantly reduced the brain levels of the estrogens 17beta-estradiol and/or estrone, and these findings indicate a correlative relationship between low estrogen and AD, perhaps suggesting that reductions in estrogens can increase AD vulnerability, specifically in women. Several retrospective and prospective studies have seemed to indicate a protective role of estrogen; hormone therapy (HT) users exhibited significantly reduced risk of dementia compared with non-users (Paganini-Hill and Henderson, 1994, 1996; Tang et al., 1996; Kawas et al., 1997; Zandi et al., 2002), with increased benefits associated with prolonged use (Zandi et al., 2002). Our team used middle-aged(9- to 12-month-old)female Sprague-Dawley (SD) rats to study the effect of estrogen replacement therapy (ERT) on white matter and found that ERT improved the spatial learning capacity and increased the myelin sheath volume of the white matter in middle-aged ovariectomized (OVX) rats (Luo et al., 2016). Although estrogen might be one important factor for the different effects observed in the current study, the exact reasons for the differences are unknown and need to be further investigated.

In conclusion, there are sex-specific differences in the white matter and myelinated fibers in the white matter in AD mice. The low values for the white matter volume and the relevant parameters of the myelinated fibers in the white matter in female AD mice might be important structural bases for the high prevalence and incidence of female AD. Running exercise could more effectively delay the decline in spatial learning and memory abilities and delay the changes of the myelinated fibers in the white matter in female transgenic mice with early AD than in male transgenic mice. In addition, running exercise could decrease the sex-specific differences in the white matter and the myelinated fibers in the white matter in AD mice. Taken together, our results might provide an important scientific basis for understanding the gender differences in AD and could provide a more appropriate starting point for the future search for preventative measures and treatments for AD.

## Author contributions

CZ and YT designed the experiments. YZ, LJ, LZ, YL, QX, and LC carried out the animal experiments. CZ, FC, YZ, LJ, and LZ analyzed the experimental results. CZ wrote the manuscript.

### Conflict of interest statement

The authors declare that the research was conducted in the absence of any commercial or financial relationships that could be construed as a potential conflict of interest.

## References

[B1] Adlard P. A. Perreau V. M. Pop V. Cotman C. W. (2005). Voluntary exercise decreases amyloid load in a transgenic model of Alzheimer's disease. J. Neurosci. 25, 4217–4221. 10.1523/JNEUROSCI.0496-05.2005 15858047 PMC6725122

[B2] Alzheimer's Association (2016). Alzheimer' disease facts and figures. Alzheimers Dement. 12, 459–509. 10.1016/j.jalz.2016.03.001 27570871

[B3] Andersen K. Launer L. J. Dewey M. E. Letenneur L. Ott A. Copeland J. R.. (1999). Gender differences in the incidence of AD and vascular dementia: the EURODEM studies. EURODEM Incidence Research Group. Neurology 53, 1992–1997. 10.1212/WNL.53.9.1992 10599770

[B4] Barnes L. L. Wilson R. S. Bienias J. L. Schneider J. A. Evans D. A. Bennett D. A. (2005). Sex differences in the clinical manifestations of Alzheimer disease pathology. Arch. Gen. Psychiatry 62, 685–691. 10.1001/archpsyc.62.6.685 15939846

[B5] Bartzokis G. (2004). Age-related myelin breakdown: a developmental model of cognitive decline and Alzheimer's disease. Neurobiol. Aging 25, 5–18. 10.1016/j.neurobiolaging.2003.03.001 14675724

[B6] Bartzokis G. Cummings J. L. Sultzer D. Henderson V. W. Nuechterlein K. H. Mintz J. (2003). White matter structural integrity in healthy aging adults and patients with Alzheimer disease: a magnetic resonance imaging study. Arch. Neurol. 60, 393–398. 10.1001/archneur.60.3.393 12633151

[B7] Bartzokis G. Lu P. H. Mintz J. (2007). Human brain myelination and amyloid beta deposition in Alzheimer's disease. Alzheimers Dement. 3, 122–125. 10.1016/j.jalz.2007.01.019 18596894 PMC2442864

[B8] Benes F. M. Farol P. A. Majocha R. E. Marotta C. A. Bird E. D. (1991). Evidence for axonal loss in regions occupied by senile plaques in Alzheimer cortex. Neuroscience 42, 651–660. 10.1016/0306-4522(91)90034-L 1956514

[B9] Bouldin E. D. Andresen E. (2010). Caregiving Across the United States: Caregivers of Persons with Alzheimer's Disease or Dementia in 8 States and the District of Columbia. Data from the 2009 & 2010 Behavioral Risk Factor Surveillance System. Washington, DC: Alzheimer's Association.

[B10] Bove R. Secor E. Chibnik L. B. Barnes L. L. Schneider J. A. Bennett D. A.. (2014). Age at surgical menopause influences cognitive decline and Alzheimer pathology in older women, Neurology 82, 222–229. 10.1212/WNL.0000000000000033 24336141 PMC3902759

[B11] Bozzali M. Falini A. Franceschi M. Cercignani M. Zuffi M. Scotti G.. (2002). White matter damage in Alzheimer's disease assessed *in vivo* using diffusion tensor magnetic resonance imaging. J. Neurol. Neurosurg. Psychiatry 72, 742–746. 10.1136/jnnp.72.6.742 12023417 PMC1737921

[B12] Bronge L. Bogdanovic N. Wahlund L. O. (2002). Postmortem MRI and histopathology of white matter changes in Alzheimer brains. A quantitative, comparative study. Dement. Geriatr. Cogn. Disord. 13, 205–212. 10.1159/000057698 12006730

[B13] Brun A. Englund E. (1986). A white matter disorder in dementia of the Alzheimer type: a pathoanatomical study. Ann. Neurol. 19, 253–262. 10.1002/ana.410190306 3963770

[B14] Burke S. L. Hu T. Fava N. M. Li T. Rodriguez M. J. Schuldiner K. L. (2018). Sex differences in the development of mild cognitive impairment and probable Alzheimer's disease as predicted by hippocampal volume or white matter hyperintensities. J. Women Aging 10, 1–25. 10.1080/08952841.2018.1419476 PMC6039284 29319430

[B15] Cahill L. (2006). Why sex matters for neuroscience. Nat. Rev. Neurosci. 7, 477–484. 10.1038/nrn1909 16688123

[B16] Chen G. Chen K. S. Knox J. Inglis J. Bernard A. Martin S. J.. (2000). A learning deficit related to age and beta-amyloid plaques in a mouse model of Alzheimer's disease. Nature 408, 975–979. 10.1038/35050103 11140684

[B17] Cho J. Y. Hwang D. Y. Kang T. S. Shin D. H Hwang J. H. Lim C. H.. (2003). Use of NSE/PS2m-transgenic mice in the study of the protective effect of exercise on Alzheimer's disease. J. Sports Sci. 21, 943–951. 10.1080/0264041031000140365 14626374

[B18] Cotman C. W. Berchtold N. C. (2007). Physical activity and the maintenance of cognition: learning from animal models. Alzheimers. Dement. 3, S30–S37. 10.1016/j.jalz.2007.01.013 19595972

[B19] Gallart-Palau X. Lee B. S. Adav S. S. Qian J. Serra A. Park J. E.. (2016). Gender differences in white matter pathology and mitochondrial dysfunction in Alzheimer's disease with cerebrovascular disease. Mol. Brain 9:27. 10.1186/s13041-016-0205-7 26983404 PMC4794845

[B20] Gundersen H. J. Bendtsen T. F. Korbo L. Marcussen N. Møller A. Nielsen K.. (1988). Some new, simple and efficient stereological methods and their use in pathological research and diagnosis. APMIS 96, 379–394. 10.1111/j.1699-0463.1988.tb05320.x 3288247

[B21] Gundersen H. J. G. (1977). Notes on the estimation of the numerical density of arbitrary profiles: the edge effect. J. Microsc. 111, 219–223. 10.1111/j.1365-2818.1977.tb00062.x

[B22] Hirsch C. (2013). ACP Journal Club. A 12-month, in-home exercise program delayed functional deterioration in Alzheimer disease. Ann. Intern. Med. 159:JC10. 10.7326/0003-4819-159-4-201308200-02010 24026274

[B23] Huang H. Fan X. Weiner M. Martin-Cook K. Xiao G. Davis J.. (2012). Distinctive disruption patterns of white matter tracts in Alzheimer's disease with full diffusion tensor characterization. Neurobiol. Aging 33, 2029–2045. 10.1016/j.neurobiolaging.2011.06.027 21872362 PMC3227739

[B24] Kavcic V. Ni H. Zhu T. Zhong J. Duffy C. J. (2008). White matter integrity linked to functional impairments in aging and early Alzheimer's disease. Alzheimers. Dement. 4, 381–389. 10.1016/j.jalz.2008.07.001 19012862 PMC2653423

[B25] Kawas C. Resnick S. Morrison A. Brookmeyer R. Corrada M. Zonderman A.. (1997). A prospective study of estrogen replacement therapy and the risk of developing Alzheimer's disease: the Baltimore Longitudinal Study of aging. Neurology 48, 1517–1521. 10.1097/00006254-199711000-00020 9191758

[B26] Kruggel F. Masaki F. Solodkin A. (2017). Analysis of longitudinal diffusion-weighted images in healthy and pathological aging: an ADNI study. J. Neurosci. Methods 278, 101–111. 10.1016/j.jneumeth.2016.12.020 28057473

[B27] Laurin D. Verreault R. Lindsay J. MacPherson K. Rockwood K. (2001). Physical activity and risk of cognitive impairment and dementia in elderly persons. Arch. Neurol. 58, 498–504. 10.1001/archneur.58.3.498 11255456

[B28] Liu C. C. Liu C. C. Kanekiyo T. Xu H. Bu G. (2013). Apolipoprotein E and Alzheimer disease: risk, mechanisms and therapy. Nat. Rev. Neurol. 9, 106–118. 10.1038/nrneurol.2012.263. 23296339 PMC3726719

[B29] Lobo A. Launer L. J. Fratiglioni L. Andersen K. Di Carlo A. Breteler M. M. (2000). Prevalence of dementia and major subtypes in Europe: a collaborative study of population-based cohorts. Neurologic diseases in the Elderly Research Group. Neurology 54, S4–S9. 10854354

[B30] Luo Y. Xiao Q. Chao F. He Q. Lv F2 Zhang L.. (2016). 17β-estradiol replacement therapy protects myelin sheaths in the white matter of middle-aged female ovariectomized rats: a stereological study, Neurobiol. Aging 47, 139–148. 10.1016/j.neurobiolaging.2016.07.023 27592282

[B31] Marx J. (2005). Alzheimer's disease. Play and exercise protect mouse brain from amyloid buildup. Science 307:1547. 10.1126/science.307.5715.1547 15761131

[B32] Mazure C. M. Swendsen J. (2016). Sex differences in Alzheimer's disease and other dementias. Lancet Neurol. 15, 451–452. 10.1016/S1474-4422(16)00067-3 26987699 PMC4864429

[B33] Mielke M. M. Vemuri P. Rocca W. A. (2014). Clinical epidemiology of Alzheimer's disease: assessing sex and gender differences. Clin. Epidemiol. 6, 37–48. 10.2147/CLEP.S37929 24470773 PMC3891487

[B34] Morris R. (1984). Developments of a water-maze procedure for studying spatial learning in the rat. J. Neurosci. Methods 11, 47–60. 10.1016/0165-0270(84)90007-4 6471907

[B35] Mosconi L. Berti V. Quinn C. McHugh P. Petrongolo G. Varsavsky I.. (2017). Sex differences in Alzheimer risk: brain imaging of endocrine vs chronologic aging. Neurology 89, 1382–1390. 10.1212/WNL.0000000000004425 28855400 PMC5652968

[B36] Musicco M. (2009). Gender differences in the occurrence of Alzheimer's disease. Funct. Neurol. 24, 89–92. 19775536

[B37] Nichol K. Deeny S. P. Seif J. Camaclang K. Cotman C. W. (2009). Exercise improves cognition and hippocampal plasticity in APOE epsilon4 mice. Alzheimers. Dement. 5, 287–294. 10.1016/j.jalz.2009.02.006 19560099 PMC4105011

[B38] Nyengaard J. R. Gundersen H. J. G. (1992). The isector: a simple and direct method for generating isotropic, uniform random sections from small specimens. J. Microsc. 165, 427–431.

[B39] Paganini-Hill A. Henderson V. W. (1994). Estrogen deficiency and risk of Alzheimer's disease in women. Am. J. Epidemiol. 140, 256–261. 8030628 10.1093/oxfordjournals.aje.a117244

[B40] Paganini-Hill A. Henderson V. W. (1996). Estrogen replacement therapy and risk of Alzheimer disease. Arch. Intern. Med. 156:2213–2217. 10.1001/archinte.1996.00440180075009 8885820

[B41] Pérez C. A. Cancela Carral J. M. (2008). Benefits of physical exercise for older adults with Alzheimer's disease. Geriatr. Nurs. 29, 384–391. 10.1016/j.gerinurse.2007.12.002 19064136

[B42] Peters A. (2009). The effects of normal aging on myelinated nerve fibers in monkey central nervous system, Front. Neuroanat. 3:11. 10.3389/neuro.05.011.2009 19636385 PMC2713738

[B43] Phung T. K. Waltoft B. L. Laursen T. M. Settnes A. Kessing L. V. Mortensen P. B.. (2010). Hysterectomy, oophorectomy and risk of dementia: a nationwide historical cohort study. Dement. Geriatr. Cogn. Disord. 30, 43–50. 10.1159/000314681 20689282

[B44] Rocca W. A. Bower J. H. Maraganore D. M. Ahlskog J. E. Grossardt B. R. de Andrade M.. (2007). Increased risk of cognitive impairment or dementia in women who underwent oophorectomy before menopause. Neurology 69, 1074–1083. 10.1212/01.wnl.0000276984.19542.e6 17761551

[B45] Roher A. E. Weiss N. Kokjohn T. A. Kuo Y. M. Kalback W. Anthony J.. (2002). Increased A beta peptides and reduced cholesterol and myelin proteins characterize white matter degeneration in Alzheimer's disease. Biochemistry 41, 11080–11090. 10.1021/bi026173d 12220172

[B46] Scheltens P. Barkhof F. Leys D. Wolters E. C. Ravid R. Kamphorst W. (1995). Histopathologic correlates of white matter changes on MRI in Alzheimer's disease and normal aging. Neurology 45, 883–888. 10.1212/WNL.45.5.883 7746401

[B47] Sjöbeck M. Haglund M. Englund E. (2005). Decreasing myelin density reflected increasing white matter pathology in Alzheimer's disease—a neuropathological study. Int. J. Geriatr. Psychiatry 20, 919–926. 10.1002/gps.1384 16163742

[B48] Sjöbeck M. Haglund M. Englund E. (2006). White matter mapping in Alzheimer's disease: a neuropathological study. Neurobiol. Aging 27, 673–680. 10.1016/j.neurobiolaging.2005.03.007 15894407

[B49] Smith C. D. Snowdon D. A. Wang H. Markesbery W. R. (2000). White matter volumes and periventricular white matter hyperintensities in aging and dementia. Neurology 54, 838–842. 10.1212/WNL.54.4.838 10690973

[B50] Tang M. X. Jacobs D. Stern Y. Marder K. Schofield P. Gurland B.. (1996). Effect of oestrogen during menopause on risk and age at onset of Alzheimer's disease. Lancet 348, 429–432. 8709781 10.1016/S0140-6736(96)03356-9

[B51] Tang Y. Nyengaard J. R. (1997). A stereological method for estimating the total length and size of myelin fibers in human brain white matter. J. Neurosci. Meth. 73, 193–200. 10.1016/S0165-0270(97)02228-0 9196291

[B52] Tang Y. Nyengaard J. R. Pakkenberg B. Gundersen H. J. (1997). Age-induced white matter changes in the human brain: a stereological investigation. Neurobiol. Aging 18, 609–615. 10.1016/S0197-4580(97)00155-3 9461058

[B53] Tang Y. Nyengaard J. R. Pakkenberg B. Gundersen H. J. G. (2003). Stereology of neuronal connections (myelinated fibers of white matter and synapses of neocortex) in human brain. Image Anal. Stereol. 22, 171–182. 10.5566/ias.v22.p171-182

[B54] Wang D. S. Bennett D. A. Mufson E. J. Mattila P. Cochran E. Dickson D. W. (2004). Contribution of changes in ubiquitin and myelin basic protein to age-related cognitive decline. Neurosci. Res. 48, 93–100. 10.1016/j.neures.2003.10.002 14687885

[B55] Wimo A. Prince M. (2010). World Alzheimer's Disease International. Alzheimer Report 2010: The Global Economic Impact of Dementia, 1–56.

[B56] Yu F. Kolanowski A. M. Strumpf N. E. Eslinger P. J. (2006). Improving cognition and function through exercise intervention in Alzheimer's disease. J. Nurs. Scholarsh. 38, 358–365. 10.1111/j.1547-5069.2006.00127.x 17181084

[B57] Yuede C. M. Zimmerman S. D. Dong H. Kling M. J. Bero A. W. Holtzman D. M.. (2009). Effects of voluntary and forced exercise on plaque deposition, hippocampal volume, and behavior in the Tg2576 mouse model of Alzheimer's disease. Neurobiol. Dis. 35, 426–432. 10.1016/j.nbd.2009.06.002 19524672 PMC2745233

[B58] Zandi P. P. Carlson M. C. Plassman B. L. Welsh-Bohmer K. A. Mayer L. S. Steffens D. C.. (2002). Hormone replacement therapy and incidence of Alzheimer disease in older women: the Cache County study. JAMA 288, 2123–2129. 10.1001/jama.288.17.2123 12413371

[B59] Zhang L. Chao F. L. Luo Y. M. Xiao Q. Jiang L. Zhou C. N. (2016). Exercise prevents cognitive function decline and demyelination in the white matter of APP/PS1transgenic AD mice. Curr. Alzheimer Res. 14, 645–655. 10.2174/1567205014666161213121353. 27978791

